# Could Wealth Make Religiosity Less Needed for Subjective Well-Being? A Dual-Path Effect Hypothesis of Religious Faith Versus Practice

**DOI:** 10.3389/fpsyg.2020.01636

**Published:** 2020-07-23

**Authors:** Xiaofang Zheng, Mengjiao Song, Hao Chen

**Affiliations:** ^1^Department of Psychological Sciences, Purdue University, West Lafayette, IN, United States; ^2^Department of Educational Psychology, University of Wisconsin-Madison, Madison, WI, United States; ^3^Department of Social Psychology, Zhou Enlai School of Government, Nankai University, Tianjin, China

**Keywords:** subjective well-being, religious faith, religious practice, income, GDP, World Value Survey

## Abstract

Religiosity is important for religious people to maintain their subjective well-being (SWB). We propose a dual-path effect hypothesis to explore different working mechanisms of religious faith and practice on benefiting people’s SWB. Religious faith can promote SWB mainly via an intrinsic meaning-making path although religious practice can promote SWB via both an intrinsic meaning-making path and an extrinsic capital-accumulating path. If the dual-path effect hypothesis stands, then the role of religious practice in influencing SWB should be partly substituted by good economic status, but the role of religious faith should not. Then, only the effect of religious practice would be moderated by wealth. Results show that people’s individual income and national GDP have significant moderating effects on the relationship between religious practice and SWB, but they had no moderating effect on the association between religious faith and SWB, indicating wealth could be an alternative source of accumulating capital and social resources between religious practice and SWB. Results provide important evidence for the dual-path effect hypothesis. The findings uniquely contribute to the literature of religiosity, SWB, and their connections with wealth. Implications for future research are also discussed.

## Introduction

Numerous studies demonstrate that religion is a powerful buffer for humans against fear, sadness, anxiety, and other suffering from the outer world and is of great help in maintaining subjective well-being (SWB) (e.g., Smith et al., 2003; Ryff et al., 2004; Harding et al., 2005; Inglehart, 2010; Diener et al., 2011; Shiah et al., 2015). Meanwhile, cross-sectional studies show that people in richer and more developed nations are less likely to be religious than those in poorer nations (e.g., Barber, 2012; Fahmy, 2018); for an individual, a higher income tends to discourage religiosity (e.g., Lipford and Tollison, 2003). What is more, longitudinal studies find that religiosity decreases with the increase in economic growth (Barro and Mitchell, 2004; McCleary and Barro, 2006) as well as with the increase in individuals’ income (Herzer and Strulik, 2017). It seems religiosity is less needed for richer people and residents in richer nations. If people gain greater SWB from religion, why is religion less needed for richer people or residents in richer nations? To our knowledge, there is no theory accounting for this phenomenon from the perspective of the different working mechanisms of religious faith and practice. Here, we fill this gap and propose a dual-path effect hypothesis to illustrate the different functions of religious faith and practice on SWB while taking the condition of individual and national wealth into account.

## Literature Review

### Religious Faith Versus Practice on SWB

Religions are defined by a shared ideology and worldview that addresses problems such as the nature of existence. Members typically perform daily moral practices as well as wider practices, customs, and rituals (Kimball, 2002). Religions of many sects provide believers with sound faith systems that bring a sense of trustworthiness and sources of comfort and encourage normative activities of the sacred or divine that strengthen members’ identification (e.g., McIntosh, 1995; Pargament, 2002; Silberman, 2003, 2005; Emmons, 2005; Park, 2005). Studies show that religion seems to promote SWB through promising life after death (Ferriss, 2002; Vail et al., 2010), existential meaning (e.g., Eckersley, 2007), and providing social support and cooperation for believers (Eliassen et al., 2005; Lim and Putnam, 2009).

Religiosity can be described as including “various dimensions associated with religious faiths and involvement” (Bergan and McConatha, 2001, p. 24). Specifically, religious faith and practice are two important dimensions that could function differently (e.g., Joshanloo and Weijers, 2016). Religious faith might promote well-being and benefit mental health (for review, see Seybold and Hill, 2001) by providing meaning and purpose for people (Myers, 2000; Galen, 2012), increasing their self-esteem (Yakushko, 2005; for reviews, Schieman et al., 2017), providing greater emotional support (Brown et al., 2004; Krause and Wulff, 2005), and helping reduce depression (e.g., Smith et al., 2003; Eliassen et al., 2005) and stress (e.g., Dezutter et al., 2010). Religious practice is also shown to promote SWB (Petersen and Roy, 1985; Idler, 1987; Ellison et al., 1989; Beeson, 2003; Law and Sbarra, 2009). For example, studies find that church attendance provides individuals with better social networks and a sense of belonging, which can reduce depression in the long term (e.g., Strawbridge et al., 2001; Parker et al., 2003). Religious institutional settings, e.g., a church, give people more sense of belonging and social safety nets by providing an environment where they can socially interact with others with similar value systems. Furthermore, according to Krause (2016), people who go to church often are more likely to help others and gain a higher sense of self-esteem that, in turn, tend to have better satisfaction for their own health.

Theories and frameworks were proposed to clarify the complicated working mechanism of religion. One of the most widespread frameworks, named the intrinsic–extrinsic framework by Allport and Ross (1967), states that people with extrinsic motivation *use* their religion, whereas people with the intrinsic motivation *live* their religion. The extrinsic religious orientation was “strictly utilitarian; useful for the self in granting safety, social standing, solace, and endorsement of one’s way of life” (Allport, 1966, p. 455). People who are extrinsically orientated instrumentally use religion for their own ends, for example, to participate in a powerful in-group (Genia and Shaw, 1991), to build social networks, to provide security, or even to gain social status (Allport and Ross, 1967). In contrast, intrinsically oriented persons find their master motivation in religion while treating others as of less ultimate importance (Allport, 1966). For intrinsic-oriented religious people, religion provides a system of beliefs and guides for seeking meanings and purposes in life (Park, 2007). As stated by Allport (1966), the intrinsic religious faith was “oriented toward a transcendent unification of being, takes seriously the commandment of brotherhood, and strives to transcend all self-centered needs” (p. 455).^1^ Some studies find that intrinsic religious orientation is positively related with various aspects of SWB (e.g., Watson et al., 1988; Kass et al., 1991; Genia, 1998; Byrd et al., 2000). Extrinsic religious orientation was shown to be involved in fewer but more complex relationships with SWB in previous research. Some studies showed a negative relationship (e.g., Genia, 1998), and other studies showed that extrinsic religious orientation has a positive but weaker effect on SWB compared with the intrinsic religious orientation (e.g., Aghababaei and Błachnio, 2014).

To clarify the different working mechanism of religious faith and practice influencing SWB with reference to the concepts of extrinsic and intrinsic religious orientation, we propose a dual-path effect hypothesis: There is an intrinsic meaning-making path and an extrinsic capital-accumulating path via which religiosity could promote SWB; although intrinsic religious faith can boost SWB mainly via an intrinsic meaning-making path, religious practice can promote SWB via both paths.

Religious faith, such as the importance of God in one’s mind, can influence SWB mostly in an intrinsic-orientation way. Religious faith could help people find meaning in life (e.g., Baumeister, 1991), and perceiving life as meaningful was shown to be associated with greater well-being (e.g., Reker et al., 1987; Zika and Chamberlain, 1992). According to the core of Allport’s extrinsic versus intrinsic belief distinction, the intrinsic religious beliefs that are inside one’s mind might not directly result in any benefits from accumulating any social capital but could, to some extent, answer people’s questions about life and existence and impose a sense of control as well as stability on the flux of life (e.g., Baumeister, 1991; Whitson and Galinsky, 2008; Kay et al., 2010; Stephens et al., 2013). In contrast, religious practice could influence SWB by both the intrinsic meaning-making path and the extrinsic capital-accumulating paths. People could attend church to find answers for questions in life or to accumulate social networks (Sacerdote and Glaeser, 2001; Krause, 2011).

### The Moderating Effect of Wealth

Wealth is important to people’s lives as it enables people to acquire resources and meet their various needs. Research shows that wealth influences people’s SWB at both micro levels, e.g., individual income (e.g., Stevenson and Wolfers, 2008; Kahneman and Deaton, 2010; Sacks et al., 2010, 2013; Diener et al., 2013), and macro levels, e.g., national GDP per capita (e.g., Sacks et al., 2010; Bartolini and Sarracino, 2014). People with worse economic status may be more vulnerable to their environment. For instance, lower income could possibly result in fewer opportunities to have a higher level of education (e.g., Aryee et al., 1999), exert environmental control, and access external resources in order to cope with diverse circumstances (e.g., Cummins, 2000). Even worse, poor individuals may have insufficient nutrition and medical care, which could lead to more disease and disability (e.g., Cummins, 2000). As such, wealth can define people’s standard of life and even their social standing (Wang et al., 2020). Situations are similar for people in poor countries. People in low-income countries have more than twice the rate of depressive symptoms, compared with their United States. counterparts, possibly due to more expensive basic health care services and limited resources (Chen and Wang, 2016). By contrast, people with better economic status are more likely to avoid these situations. Wealth also plays an important role in valued social resources. Many studies demonstrate that wealth enables people to pursue their goals and need less from social networks (Vohs et al., 2006; Kraus et al., 2012). In this sense, wealth can promote SWB by accumulating capital and social resources, which is in line with how religious practice works on SWB via the extrinsic capital-accumulation path in our dual-path effect hypothesis.

On the other hand, there seems to be limited evidence proving that the richer an individual is, the more meaning in life he/she finds. Meaning in life is an important aspect of eudaimonic well-being, which emerges through the fulfillment of intrinsic motivation (e.g., Ryan and Deci, 2001), whereas limited evidence shows that wealth seems not to be related with meaning in life. For example, Graham (2011) used within- and cross-country data and found that wealth is not significantly correlated with life purpose (measured by the question “Do you feel your life has purpose or meaning?”).

Based on the discussions above, the role of religious practice in influencing SWB could be partly substituted by good economic status, but the role of religious faith could not. Religious practice might compete with other sources that can meet the same needs of people (Iannaccone and Everton, 2004), e.g., wealth. If the dual-path hypothesis is true, then only the effect of religious practice would be moderated by wealth. We hypothesized that both religious faith and practice can significantly and positively drive the change of SWB, but only the effect of religious practice can be moderated by wealth, e.g., income and GDP. In other words, for people with higher income or living in more developed nations, the same increment of religious practice might bring less increase in SWB, but the effect of religious faith could be similar. We used worldwide representative surveys and national census data over 30 years to test this hypothesis on how religious faith and practice work on SWB in the moderating effect of individual/national economic factors.

## Materials and Methods

### Data

We used the data collected by the World Values Survey (WVS) ^2^for SWB, demographic covariates, individual income, religious practice, and religious faith. WVS consists of nationally representative surveys conducted in almost 100 countries, which contain around 90% of the world’s population, 348,532 (mean age = 40.8 years; 51.48% female) subjects for 30 years (Inglehart et al., 2014). The data include individuals’ attitudes and values on political, cultural, economic, and civic beliefs, and other aspects of life. Each survey consists of a representative sample of a country’s residents aged 18 years and older. In the present study, we used data of total six waves (Wave 1: 1981–1984; Wave 2: 1990–1994; Wave 3: 1995–1998; Wave 4: 1999–2004; Wave 5: 2005–2009; Wave 6: 2010–2014). We collected data on the GDP from the World Bank and the Penn World Table.

### Measures

#### Subjective Well-Being

Some studies used a single item to measure SWB, like life satisfaction (e.g., Stevenson and Wolfers, 2008; Joshanloo and Weijers, 2016) or happiness (e.g., Francis et al., 2004; Abdel-Khalek, 2006). However, it is argued that the single measure of SWB from one dimension may not be accurate (Bartolini and Sarracino, 2014; Diener et al., 2017). To make our measurement of SWB more comprehensive, we followed the work by Greenaway et al. (2015) to composite three items in WVS as the measure for SWB.^3^ The three items were life satisfaction (“All things considered, how satisfied are you with your life?” Answer using a 10-point scale from 1 = dissatisfied to 10 = satisfied), happiness (“Taking all things together, would you say you are…” Answer using a four-point scale from 1 = very happy to 4 = not at all happy), and subjective health (“All in all, how would you describe your state of health these days?” Answer using a four-point scale from 1 = very good to 4 = poor). These items were common indicators of SWB (e.g., Andrews and Withey, 1978; Inglehart et al., 2008), which together could measure SWB in broader facets and more accurately. To composite the three items, we reversed the scores for happiness and subjective health, standardized the scores of each item, and added the standardized scores together.

#### Religious Faith

We used religious faith and religious practice as the measures for two main dimensions of individual religiosity. Religious faith was measured by the question: “How important is God in your life?” on a 10-point scale (1 = not at all important; 10 = very important). We measured in this way following former studies (e.g., Koster et al., 2009; Okulicz-Kozaryn, 2010; Hayward and Elliott, 2014; Chon, 2016).

#### Religious Practice

Religious practice was measured by the attendance frequency of religious services (e.g., Strawbridge et al., 2001; Li et al., 2016). The one-item question was “Apart from weddings and funerals, about how often do you attend religious services these days?” on an eight-point scale (1 = more than once a week, 8 = practically never attend). For the sake of convenience, we reversed the original score scale and used the reversed scale (1 = practically never attend, 8 = more than once a week) in the current study.

#### Individual Income

Individual income was measured by a single item of subjective scale on income in WVS (e.g., Jen et al., 2009; Lucas and Schimmack, 2009). Respondents answered the following question on a 10-point scale (1 = the lowest level; 10 = the highest level): “Here is a scale of incomes. We would like to know in what group your household is, counting all wages, salaries, pensions, and other incomes that come in. Just give the letter of the group your household falls into before taxes and other deductions.”

#### GDP

Data of the national annual GDP were attained from the World Bank (World Development Indicators Online^4^) for the period 1981–2014 (e.g., Diener et al., 2010; Easterlin, 2015). Those data were an extrapolation of the World Bank series used the Penn World Table (Heston et al., 2006). We used the logarithm form of the original data in the present study (e.g., Easterlin et al., 2010; Sacks et al., 2010).

#### Demographics

Previous studies show significant relationships between SWB and a range of demographics such as age (e.g., Rözer and Kraaykamp, 2013), gender (e.g., Tesch-Römer et al., 2008), education (e.g., Joshanloo and Weijers, 2016), and social class (e.g., Artazcoz et al., 2004). We took these variables into consideration and built hierarchical models to control their effects. In WVS, participants were strictly adults, between 18 and 99 years old. Gender was coded as men and women (men were coded as 1, women were coded as 2). Education was measured by a one-item question about the highest education the subject obtained (1 = inadequately completed elementary education, 8 = university with degree/higher education). Social class was the subjective social class in which people described themselves (1 = upper class, 5 = lower class). We reversed the original coding scheme of social class and used a higher score for the upper social class in the current study.

## Results

See Table 1 for sample sizes, average religious faith and practice, average individual income, and national GDP in log form (which were all standardized) of each nation. The table includes 86 countries and regions across the world. We built hierarchical mixed-effect models to test the relationship between religiosity and SWB. Taking consideration of individual differences, we allowed the intercept and the slopes of individual-level variables to vary across cultures. We used SWB as the dependent variable. Independent variables included individual religiosity (religious faith or religious practice), individual income/national GDP, and covariates (gender, age, highest education degree, and social class). See Table 2 for the result of correlations between independent variables and SWB. All independent variables were significantly associated with SWB. To test moderating effects, we added the interactions of individual religiosity and individual income into model 1 and the interactions of individual religiosity and national GDP into model 2. The models were as follows:

**TABLE 1:** Sample sizes and mean scores of countries (Standardized).

	**Sample size**	**SWB**	**Religious faith**	**Religious practice**	**Individual income**	**National GDP**
Spain	5343	0.250	–0.50	–0.25	–0.24	1.02
Sweden	3557	1.04	–1.04	–0.74	0.40	0.80
Trinidad and Tobago	1962	0.86	0.68	0.26	0.11	–1.10
Japan	5953	–0.07	–0.81	–0.29	–0.02	2.09
South Korea	5370	–1.37	–1.28	0.00	0.08	0.57
United States	7740	0.80	0.27	0.13	0.51	2.25
Armenia	1680	–1.53	–0.12	–0.12	–0.49	–1.95
Russia	7243	–1.48	–0.45	–0.51	0.11	1.41
Azerbaijan	2578	–0.38	0.58	–0.33	–0.10	–1.26
Belarus	4207	–1.71	–0.37	–0.30	0.27	–1.06
Ukraine	2836	–1.87	–0.35	–0.40	–0.26	–0.25
Estonia	2392	–1.16	–0.94	–0.67	–0.10	–0.78
Kyrgyzstan	989	–0.18	–0.30	–0.44	–0.11	0.11
Nigeria	4582	1.11	0.64	1.07	0.31	–0.63
Zimbabwe	825	–1.45	0.43	0.53	–0.58	–1.36
Ghana	1409	0.27	0.67	0.86	–0.05	–1.01
Mexico	8037	0.96	0.43	0.39	–0.13	0.77
Romania	4051	–1.20	0.42	0.12	–0.12	–0.11
Philippines	2388	0.29	0.53	0.59	–0.03	–0.13
New Zealand	2465	1.24	–0.68	–0.60	0.60	0.57
Peru	4942	–0.30	0.41	0.29	–0.47	–0.11
Rwanda	1398	–1.63	0.54	1.18	–0.52	–2.00
Pakistan	1577	–0.83	0.58	0.83	0.02	–0.08
Singapore	1954	0.57	–0.16	0.08	0.49	–0.17
Chile	5313	0.42	0.36	–0.01	0.05	–0.62
Poland	2726	–0.01	0.35	–0.31	0.01	0.32
Turkey	8042	0.13	0.47	–0.07	–0.24	0.50
China	3276	0.06	–1.12	–0.81	–0.26	1.90
Netherlands	2491	0.59	–1.00	–0.69	–0.36	0.51
Australia	4837	0.93	–0.44	–0.51	0.26	0.68
Colombia	7211	1.36	0.70	0.32	–0.18	0.09
Slovenia	1873	–0.09	–0.80	–0.30	0.11	–0.60
South Africa	13711	0.55	0.42	–0.02	0.05	0.33
Germany	4655	0.19	–0.86	–0.52	0.13	1.79
Thailand	2539	0.72	–0.41	0.44	0.24	0.17
Argentina	4784	0.43	0.19	–0.15	–0.15	0.32
Iraq	5396	–1.30	0.63	–0.27	–0.05	–0.43
Jordan	2305	–0.03	0.66	0.11	–0.12	–1.19
Brazil	4706	0.71	0.68	0.40	–0.28	1.08
Hong Kong	1893	–0.43	–0.99	–0.77	0.00	–0.09
India	12935	–0.14	0.02	0.46	–0.13	0.96
Georgia	3293	–1.43	0.28	–0.05	–0.55	–1.55
France	875	0.38	–1.04	–0.86	–0.42	1.36
Canada	3459	1.34	–0.28	–0.31	0.32	1.12
Albania	1896	–1.06	–0.27	–0.11	0.18	–1.64
Algeria	2043	–0.63	–0.60	0.05	–0.24	0.16
Bangladesh	2819	–0.35	0.58	0.80	0.03	–0.23
Bulgaria	1650	–1.48	–0.72	–0.42	0.01	–0.94
Taiwan	702	0.34	–0.55	–0.62	0.47	1.00
Cyprus	1024	0.34	–0.55	–0.62	0.47	1.00
Czech Republic	1806	–0.17	–1.54	–0.40	0.29	–0.81
Dominican Republic	322	0.74	0.66	0.34	0.03	–0.71
El Salvador	1019	1.26	0.81	0.56	0.36	–0.96
Ethiopia	1424	–1.04	0.47	0.82	0.27	–1.14
Finland	1791	0.86	–0.52	–0.50	–0.18	0.20
Guatemala	882	0.77	0.65	0.81	–1.00	–1.04
Hungary	1406	0.01	–0.24	–0.36	–0.76	–0.37
Indonesia	2546	0.32	0.58	0.60	0.39	0.38
Latvia	1070	–1.33	–0.41	–0.40	0.22	–1.29
Lithuania	835	–1.46	–0.21	0.03	0.80	–1.01
Malaysia	1189	0.74	0.09	0.90	0.57	–0.03
Mali	994	0.00	0.43	0.71	0.18	–1.60
Moldova	2760	–2.14	–0.12	–0.17	–0.16	–2.04
Morocco	1971	–0.34	0.58	0.67	–0.06	–0.41
Netherlands	2491	–0.67	0.05	–1.00	–0.36	0.51
Norway	1968	1.32	–1.00	–0.70	0.35	0.43
Puerto Rico	1717	1.48	0.66	0.67	–0.47	–0.21
Saudi Arabia	1345	1.35	0.49	–0.19	0.40	0.52
Vietnam	2325	0.15	–1.11	–0.77	0.36	–0.50
Switzerland	3383	1.47	–0.16	–0.12	0.21	0.41
Uganda	554	0.00	0.27	0.71	–0.67	–1.44
Macedonia	1607	–0.60	–0.30	–0.10	0.03	–1.64
Egypt	5714	–0.60	0.58	0.04	0.03	0.02
United Kingdom	795	1.00	–0.75	–0.60	0.75	1.40
Tanzania	1006	–0.42	0.42	0.78	–0.58	–1.13
Burkina Faso	1227	–0.48	0.44	0.75	0.31	–1.70
Uruguay	2728	0.63	–0.19	–0.76	0.05	–0.76
Venezuela	1109	1.26	0.65	0.22	–0.49	–0.03
Zambia	1078	–0.44	0.45	0.63	0.38	–1.31
Serbia	2091	–0.75	–0.76	–0.34	0.22	–0.82
Montenegro	938	–0.75	–0.79	–0.52	0.71	–2.15
Bosnia	761	–0.12	0.12	0.21	–0.03	–0.75

**TABLE 2:** Correlations of independent variables and SWB.

**Variables**	**Coefficient**
Age	−0.137***
Gender	−0.020***
Education	0.136***
Social class	0.118***
Religious faith	0.074***
Religious practice	0.050***
Individual income	0.226***
National GDP	0.070***

*p < 0.05; **p < 0.01; ***p < 0.001.

$$\mathrm{Model}\,1:\text{SWB}=\beta_{0}+\beta_{1}\,\text{Gender}+\beta_{2}\,\text{Age}+\beta_{3}\,\text{Education}+$$

$$\beta_{4}\,\mathrm{Social}\,\mathrm{Class}+\beta_{5\,}\,\mathrm{Religious}\,\mathrm{Faith}+\beta_{6}\,\mathrm{Religious}\,\mathrm{Practice}+$$

$$\beta_{7}\,\mathrm{Individual}\,\mathrm{Income}+\beta_{8}\,\mathrm{Religious}\,{\mathrm{Faith}}^{*}\,\mathrm{Individual}\,\mathrm{Income}$$

$$+\beta_{9}\,\mathrm{Religious}\,{\mathrm{Pracitice}}^{*}\,\mathrm{Individual}\,\mathrm{Income}+\varepsilon ;$$

$$\mathrm{Model}\,2:\text{SWB}=\beta_{0}+\beta_{1}\,\text{Gender}+\beta_{2}\,\text{Age}+\beta_{3}\,\text{Education}+$$

$$\beta_{4}\,\mathrm{Social}\,\mathrm{Class}+\beta_{5\,}\,\mathrm{Religious}\,\mathrm{Faith}+\beta_{6}\,\mathrm{Religious}\,\mathrm{Practice}+$$

$$\beta_{7}\,\text{GDP}+\beta_{8}\,\mathrm{Religious}\,{\mathrm{Faith}}^{*}\,\mathrm{GDP}+\beta_{9}\,\mathrm{Religious}\,{\mathrm{Pracitice}}^{*}$$

G⁢D⁢P+ε.

**FIGURE 1:**
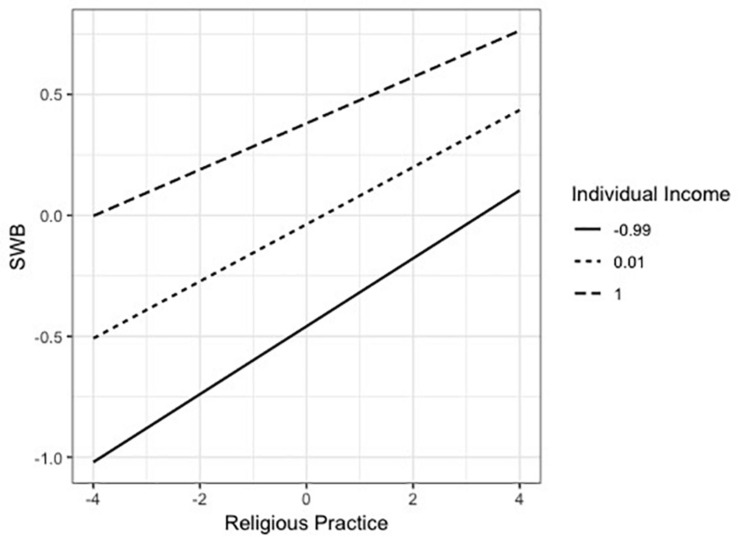
Moderating effect of individual income on the relation between religious behavior and SWB in Model 1.

Analysis and figures were conducted in R 3.6.1 (R Core Team, 2019). See Table 3 for the results of model 1. Generally, younger age (*B* = −0.020, *t* = −14.959, *p* < 0.001), higher education level (*B* = 0.118, *t* = 8.169, *p* < 0.001), the gender of male (for female, *B* = −0.054, *t* = −2.225, *p* < 0.05), and upper social class (*B* = 0.248, *t* = 12.103, *p* < 0.001) were positively associated with greater SWB. Religious faith and practice were both positively and significantly associated with SWB (for religious faith alone, *B*_1_ = 0.104, *t_1_* = 4.208, *p_1_* < 0.001; for religious practice alone, *B*_2_ = 0.118, *t_2_* = 7.552, *p_2_* < 0.001). Income also had a significant and positive effect on SWB (*B* = 0.422, *t* = 18.251, *p* < 0.001). The interaction of religious practice and individual income was significant (*B* = −0.023, *t* = −4.695, *p* < 0.001). The schematic representation of the results, as shown in Figure 1, indicate that the effects of religious practice on SWB were less for people with higher individual income compared to those with lower income.

**TABLE 3:** Hierarchical mixed-effect modeling predicting SWB (model 1).

	**b**	**S.E.**	***t***	**Sig.**
Intercept	0.777	0.088	8.865	0.000***
Age	−0.020	0.001	−14.959	0.000***
Female	−0.054	0.024	−2.225	0.026*
Education	0.118	0.014	8.169	0.000***
Social Class	0.248	0.020	12.103	0.000***
Individual Income	0.422	0.023	18.251	0.000***
Religious Faith	0.104	0.025	4.208	0.000***
Religious Practice	0.118	0.016	7.552	0.000***
Religious Faith × Individual Income	0.002	0.005	0.337	0.737
Religious Practice ×*IndividualIncome*	−0.023	0.005	−4.695	0.000***

*p < 0.05; **p < 0.01; ***p < 0.001.

**FIGURE 2:**
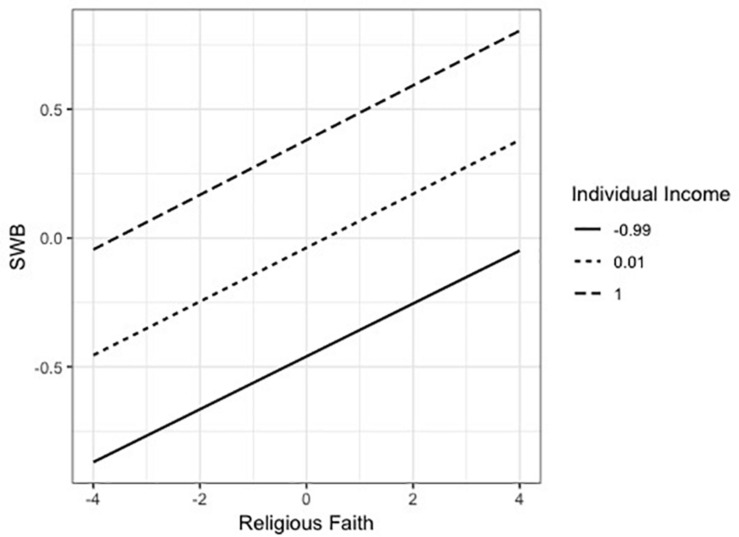
Moderating effect of individual income on the relation between religious faith and SWB in Model 1.

Yet the interaction of religious faith and individual income was not significant (*B* = 0.002, *t* = 0.337, *p* = 0.737). As shown in Figure 2, the variation of SWB by the change of religious faith was similar for people regardless of their income.

See Table 4 for the results of model 2. Age (*B* = −0.021, *t* = −14.494, *p* < 0.001), gender (for female, *B* = −0.073, *t* = −2.881, *p* < 0.01), highest education level (*B* = 0.207, *t* = 12.982, *p* < 0.001), and social class (*B* = 0.431, *t* = 16.650, *p* < 0.001) were all significant predictors of SWB. Religious faith (*B* = 0.085, *t* = 3.309, *p* < 0.01) and religious practice (*B* = 0.121, *t* = 7.149, *p* < 0.001) had significantly positive effects on SWB. National GDP also had a significant and positive effect on SWB (*B* = 0.049, *t* = 20.407, *p* < 0.001).

**TABLE 4:** Hierarchical mixed-effect modeling predicting SWB (model 2).

	**b**	**S.E.**	***t***	**Sig.**
Intercept	0.827	0.088	9.367	0.000***
Age	−0.021	0.001	−14.494	0.000***
Female	−0.073	0.025	−2.881	0.004**
Education	0.207	0.016	12.982	0.000***
Social Class	0.431	0.026	16.650	0.000***
National GDP	0.049	0.002	20.407	0.000***
Religious Faith	0.085	0.026	3.309	0.001**
Religious Practice	0.121	0.017	7.149	0.000***
Religious Faith × National GDP	−0.001	0.002	−0.541	0.589
Religious Practice × National GDP	−0.013	0.003	−4.969	0.000***

*p < 0.05; **p < 0.01; ***p < 0.001.

**FIGURE 3:**
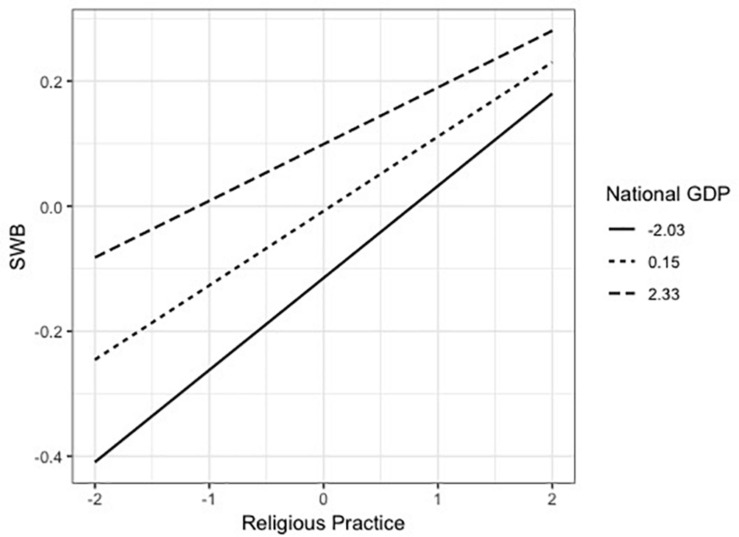
Moderating effect of national GDP on the relation between religious practice and SWB in Model 2.

**FIGURE 4:**
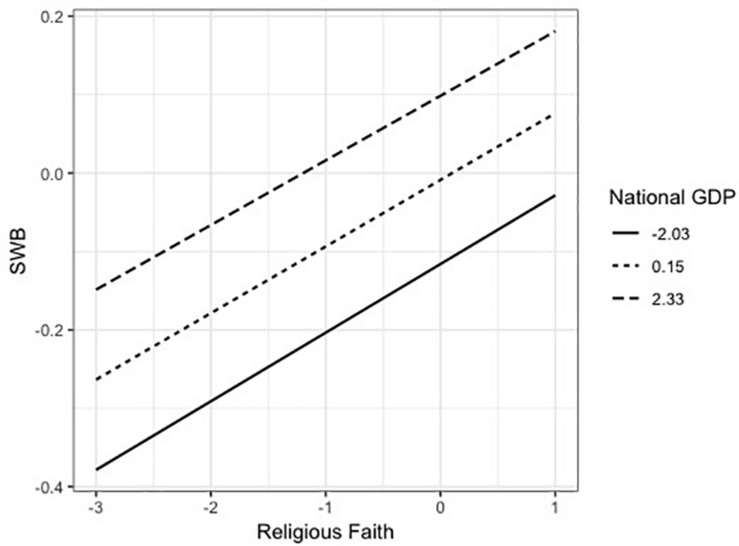
Moderating effect of national GDP on the relation between religious faith and SWB in Model 2.

The interaction effect of religious practice and GDP was significant (*B* = −0.013, *t* = −4.969, *p* < 0.001). As shown in Figure 3, for residents of nations with higher national GDP, the same increment of religious practice was associated with less increase of SWB than those for residents of nations with lower GDP. The interaction between religious faith and GDP was not significant (*B* = −0.001, *t* = −0.541, *p* = 0.589) as shown in Figure 4. This shows that the variation of SWB by the change of religious faith would be similar for residents of countries with different GDP.

## Discussion

The main purpose of the present study was to investigate the possible different working paths of religious faith and religious practice on SWB. We proposed a dual-path effect hypothesis: Religiosity could promote SWB through an intrinsic meaning-making path and an extrinsic capital-accumulating path; although religious faith could boost SWB mainly via an intrinsic meaning-making path, religious practice can promote SWB via both paths. Results show that people’s income and national GDP could moderate the relationship between an individual’s religious practice and SWB but had no significant moderating effect on the relationship between religious faith and SWB. Our results show important evidence for the dual-path effect hypothesis.

As we previously introduced, Allport and Ross (1967) first developed the idea of extrinsic and intrinsic dimensions of religiosity: the intrinsic-oriented religious people regard religion as the master motive in life, following and living their life by religious beliefs; extrinsically religious people use religion to meet various needs, for example, to gain social status. Following research has conducted many frameworks and empirical studies. Batson (1976) proposed a third religious orientation, quest orientation, which is the tendency to regard religion as a way of searching for and understanding personal meaning. Krauss and Hood (2013) also raised a more comprehensive definition that has both subjective and objective facets for a religious orientation–commitment–reflectivity circumplex model. In this paper, we propose the dual-path effect hypothesis referring to intrinsic–extrinsic orientations of religiosity to clarify the different working paths of religious faith and practice regarding promoting SWB. Better economic status could represent a better accumulation of social capital. Richer people have more access to various resources and have better opportunities to get material supports, which could result in greater SWB. Religious practice, to some extent, can boost SWB via a similar path. Many people might go to church for sociality, fulfilling social needs, seeking security and spiritual supports, or under other social pressures, which could be contributors to stable social networks (Strawbridge et al., 2001; Law and Sbarra, 2009). In this sense, the positive effect of attending religious activities could be moderated by wealth. People of higher economic status tend to have more resources and greater abilities to fulfill their demand for social needs, social standing, and so on, which makes it unnecessary for rich people to attend religious activities for greater SWB. In fact, studies did show that religious practice (e.g., attendance at religious services) was negatively associated with economic growth (Barro and McCleary, 2003; McCleary and Barro, 2006; Campante and Yanagizawa-Drott, 2015).

By contrast, religious faith indicates people’s intrinsically motivated religiosity, reflecting a spiritual need and motivation for finding meaning and purpose in life. Believing in religion and religious knowledge gives people more understanding of the meaning of life, power, and confidence to deal with the challenges and miseries in life (Emmons, 2005; Galek et al., 2015). It is less likely that the effect of faith of religion on SWB could be replaced by that of factors leading to capital accumulation. In this sense, it seems that advantages brought by wealth have nothing directly to do with the spiritual support from religion in terms of boosting SWB. Thus, the effect of religious faith on SWB would not be moderated by income and GDP, but the effect of religious practice could be. The mechanisms behind them need further investigation.

The current study also sheds light on studies about the relationship between money and religion. We tested the moderating effect of wealth on the association between religiosity and SWB using both individual income and national GDP. Our model shows that an individual’s income and national GDP are positively associated with individual’s SWB, which is consistent with former studies (e.g., Stevenson and Wolfers, 2008; Diener et al., 2013; Sacks et al., 2013). We also found that income and GDP moderated the relationship between religious practice and SWB but had no moderating effect on the relationship between religious faith and SWB. That is to say, for people with higher income or living in wealthier countries, the same increment of religious practice would bring about less increase of SWB than that for people with lower income or living in poor countries; the same increment of religious faith has no difference in increasing SWB for people whether they are poor or rich. Our study demonstrates that, in terms of buffering negative aspects of life and promoting greater SWB, the economic situation of where people live and how much money people make might compensate for the individual’s religious practice but not religious faith.

The current study provides important directions for further studies. One can follow up on our study and find more evidence for the dual-path effect hypothesis in several directions. Different sects of religion may coexist in one country. We did not specify the religious sects. Will the relationship among religiosity, economic situation, and SWB be different across religion sects? Moreover, competing mechanisms may influence the relationship between religiosity, economic situation, and SWB in different cultural and economic contexts. Other important variables that were not included could be at play. There are also limitations on the data source. We followed former studies to use one item in WVS to measure one construct. Also, the large sample size of WVS may increase the probability of Type II error. We encourage future studies to use other data sources to retest and provide more evidences for the dual-path effect hypothesis.

To sum up, our study provides important evidence for the positive relationship between religiosity and SWB by multilevel regression models using data across 86 cultures and 30 years. We contribute to the literature of religion and SWB that we propose a dual-path effect hypothesis to clarify the different working paths of religious faith and practice for promoting SWB. Results of statistical tests provide important evidence for the hypothesis. Our results indicate that people’s economic status could moderate the relationship between religious practice and SWB but had no moderating effect on the association between intrinsic religious faith and SWB. The findings about SWB help policymakers to better understand how to design and implement policies that can meet citizens’ needs. Although more evidence needs to be provided, the content and magnitude of the current study warrant further investigation into the different working mechanisms of religious faith and practice as well as the links among religiosity, economic situation, and SWB.

## Data Availability Statement

Publicly available datasets were analyzed in this study. This data can be found here: http://www.worldvaluessurvey.org/WVSContents.jsp, https://data.worldbank.org, and https://www.rug.nl/ggdc/productivity/pwt/.

## Author Contributions

All authors listed have made a substantial, direct and intellectual contribution to the work, and approved it for publication.

## Conflict of Interest

The authors declare that the research was conducted in the absence of any commercial or financial relationships that could be construed as a potential conflict of interest.

## Footnotes

1 Following Allport and Ross’s work, some studies came up with other dimensions, e.g., quest orientation by Batson (1976). Our study only focuses on the intrinsic and extrinsic orientations of religiosity.

2 http://www.worldvaluessurvey.org

3 We also built models using an aggregated indicator by life satisfaction and happiness as the measurement for SWB. The results are consistent with the results reported in the paper.

4 https://data.worldbank.org
